# Falls and functional fitness among older adults in Sub-Saharan Africa: findings from the first population-based cross-sectional study in Ghana

**DOI:** 10.1186/s12877-026-07577-6

**Published:** 2026-05-04

**Authors:** Bertha Oppong-Yeboah, Alfred Edwin Yawson, Koen Milisen, Jannique van Uffelen, Jos Tournoy

**Affiliations:** 1 Gerontology and Geriatrics Unit, Department of Public Health and Primary Care, UZ Leuven, Herestraat 49 - box 7003, Leuven, 3000 Belgium; 2 Department of Physiotherapy and Rehabilitation, University of Health and Allied Sciences, Ho, PMB 31 Ghana; 3 Department of Community Health, University of Ghana Medical School, P.O. Box GP 4236, Accra, Ghana; 4 Academic Centre for Nursing and Midwifery, Department of Public Health and Primary Care, KU Leuven, Kapucijnenvoer 7 blok g - box 7001, Leuven, 3000 Belgium; 5 Physical Activity, Sports & Health Research Group, Department of Movement science, KU Leuven, Tervuursevest 101 - box 1500, Leuven, 3001 Belgium; 6 Department of Geriatric Medicine, UZ Leuven, Leuven, Belgium

**Keywords:** Falls, Functional fitness, Older adults, Ghana, Sub-Saharan Africa, Fall risk

## Abstract

**Background:**

Falls are a leading cause of injury and disability among older adults worldwide, yet data from sub-Saharan Africa remain scarce. This study aimed to estimate the prevalence of fallers, recurrent fallers, and individuals at high risk of falls; identify associated sociodemographic and health-related factors; and examine the relationship between functional fitness and fall outcomes among older adults in Ghana, a country representative of the broader sub-Saharan African context.

**Methods:**

We conducted a population-based cross-sectional study among 639 community-dwelling adults aged ≥ 60 years in urban and rural Ghana, using multi-stage sampling. Fall history over the past 12 months was assessed through self-report, and fall risk was evaluated using the 12-item Fall Risk Questionnaire. Functional fitness was measured using eight performance tests adapted from the Senior Fitness Test and Short Physical Performance Battery. Associations between fall outcomes, sociodemographic and health factors, and fitness measures were analysed using Poisson regression with robust variance and negative binomial regression models.

**Results:**

The prevalence of fallers, recurrent fallers, and high fall risk was 24.6%, 10.3%, and 30.2%, respectively. Female sex was strongly associated with both fallers and recurrent fallers. Rural residence was significantly associated with recurrent fallers. Moderate pain and high sedentary behaviour, were also linked to fallers. Fall risk scores were strongly associated with reported fall events, with psychological and strength-related items contributing most to fall outcomes.

Poor lower limb strength, assessed using the five-times sit-to-stand test, was significantly associated with recurrent fallers (PR = 1.08; 95% CI: 1.04-1.13), while declines in any fitness domain were associated with elevated fall risk.

**Conclusion:**

Falls are common among older adults in Ghana, with risk influenced by sex, residence, and functional status. Functional fitness plays a central role in fall risk, underscoring the value of targeted interventions to promote safe and healthy ageing in sub-Saharan Africa.

**Supplementary Information:**

The online version contains supplementary material available at 10.1186/s12877-026-07577-6.

## Background

Falls are a major public health issue among older adults, linked to injury, loss of independence, disability, mortality, and substantial healthcare costs [1]. The World Health Organization (WHO) reported in 2007 that 28–35% of adults aged 65 and above fall each year, rising to 32–42% among those over 70 [2], while a recent review estimated a global prevalence of 26.5% [3].

In high-income countries, falls have been extensively studied, generating a large body of evidence on its prevalence, risk factors, and relation with other geriatric syndromes such as frailty and sarcopenia [4–9]. This knowledge has informed fall prevention guidelines since the early 2000s [10]. However, despite decades of research and the availability of effective fall prevention interventions, a recent meta-analysis found no significant decline in fall rates among community-dwelling older adults in Europe [11]. This likely reflects persistent challenges in integrating these interventions into routine clinical practice, community settings, and daily life, which limits their overall impact [12, 13]. As a result, falls remain a pressing public health concern, even in well-resourced environments.

In contrast, research on the health of older adults in sub-Saharan Africa, and on falls specifically, remains limited, despite clear evidence of a demographic shift towards an ageing population in the region [14, 15]. A recent review of the available literature found that only 5 out of 45 countries in sub-Saharan Africa had conducted studies on falls among community-dwelling older adults; Ghana had not conducted such a study [16]. In these five countries, reported fall prevalence rates ranged from 20.8% to 55.1%, and recurrent fall rates were between 11.0% and 24.6%, highlighting the scale of the issue in sub-Saharan Africa. This paucity of primary studies describing fall risk factors in most countries across the region, combined with the variability in quality and scope of the available studies, means there is insufficient data upon which robust context-specific fall prevention strategies could be based.

Functional fitness describes the level of physical performance and functioning of adults and is defined as the physiologic capacity to perform normal everyday activities safely and independently without undue fatigue [17]. It is considered a composite of several aspects of fitness. Among older adults, commonly assessed components include muscle strength, walk speed, flexibility, cardiorespiratory endurance, and balance [18]. Impairments in these areas, particularly balance, have consistently been identified as risk factors for falls in systematic reviews [4–7]. Muscle strength and gait speed are also frequently reported as significant contributors. Despite their importance, these functional fitness components have been underexplored in sub-Saharan Africa, and existing studies on falls in the region have overlooked them.

Ghana represents a prototypical lower-middle-income country (LMIC) in sub-Saharan Africa, making it a strategically important and highly generalisable setting for falls research. Its balanced urban-rural population distribution, diverse sociocultural context, and mixed public-private healthcare system, mirror the structural realities faced across the region. Like many LMICs in the region, Ghana is undergoing a demographic and epidemiological transition, with persistent infectious diseases alongside a growing burden of non-communicable and age-related conditions [19]. Notably, Ghana has one of the highest proportions of adults aged 60 years and above in the region, currently at 6.5% and projected to double to 11.6% by 2050 [15, 20]. Furthermore, Ghana’s active participation in global health surveillance systems, such as the WHO Study on global AGEing and adult health (SAGE), and her frequent role as a pilot site for public health initiatives, strengthen the regional relevance and generalisability of findings derived from this setting [21].

The present study therefore aims to provide a comprehensive assessment of falls and fall risk among community-dwelling older adults in Ghana; a representative sub-Saharan Africa setting, employing recognised measures of falls and functional fitness. Specifically, we seek to:

1. Estimate the prevalence of fallers, recurrent fallers, and high fall risk;
2. Examine the association between assessed fall risk and falls;
3. Identify sociodemographic and health-related factors associated with falls;
4. Assess the relationship between functional fitness components and fall outcomes.

## Methods

### Study design and sampling

This population-based cross-sectional study employed multi-stage sampling to ensure representation of both urban and rural older adults in Ghana. The Greater Accra Region and Eastern Region were purposively selected out of sixteen regions based on census data, as they had the highest density of older adults in urban and rural areas, respectively [20]. From these two regions, nine administrative districts were randomly selected through balloting.

### Participant recruitment

In the selected districts, participants were recruited through local community-based organisations and older adult groups typically affiliated with religious institutions, pensioner associations, or neighbourhood-based. Initial contact was made with the group leaders and the study rationale, procedures, and eligibility criteria were explained to them. With agreement to access their members, we followed up by either attending association meetings to explain the study rationale, procedures, and eligibility criteria to the members, or through phone calls, or posting information sheets in their WhatsApp groups. After these contacts, a suitable date was set for data collection for each group. Written informed consent was obtained prior to participation. For individuals with low literacy, a literate witness assisted with the consent process.

### Eligibility criteria

Participants were eligible if they were aged sixty or older, resided in the selected communities, and were able to walk independently (with or without a walking aid). Exclusion criteria included severe cognitive, or communication impairment observed during the intake process, and unstable medical conditions, that would prevent safe participation in the physical performance testing. Questions concerning a history of chest pain with or without activity, loss of balance due to dizziness, loss of consciousness or a fracture within the last six months were used to ascertain their medical stability.

### Data collection procedure

The study received approval from the Ethics Committee of KU/UZ Leuven, Belgium (S67894), and formal clearance from the Ethics and Protocol Review Committee of the College of Health Sciences, University of Ghana (CHS-Et/M.3-P 4.1/2023-24).

Data were collected from March to August 2024 by trained research assistants who were qualified physiotherapists and exercise scientists. Training covered standardized protocols for administering questionnaires and physical tests, and verbal translation of questionnaires into Twi and Ga, the predominant local languages. As native speakers of these languages, training focused on ensuring consistency in word choice and prompts. Inter-rater reliability was assessed among team members for the physical performance tests, yielding high intra-class correlation coefficients (ICC = 0.85–0.99). Data were collected via face-to-face interviews in English or one of the local languages. Physical performance tests were conducted in community centres or participants’ homes, with efforts to ensure consistent and safe environments. All testing areas had even, hard, and non-slippery floors, including ceramic tiles, screed, or terrazzo. Group assessments followed a structured circuit-style protocol lasting 40–60 min, which was pre-tested for feasibility. A description of the circuit assessment and all data collection measures are available in the attached supplementary material (Additional file 1). Data were initially recorded on paper and later entered into the REDCap system. A random 10% data entry audit revealed a very low error rate (< 0.5%), indicating high data entry quality.

### Measures

#### Fall outcomes

A fall was defined in accordance with WHO standards as ‘an event that results in a person coming to rest inadvertently on the ground, floor, or other lower level’ [2]. This was explained to the participants to ensure that events without landing or near-falls were not misclassified as falls. The prevalence of fallers was assessed based on a “Yes” response to the question: “Have you fallen in the last 12 months?” Recurrent fallers were defined as reporting more than one fall in response to the question: “How many falls have you had in the last 12 months?” Fall risk for all participants was assessed using the 12-item, self-rated Fall Risk Questionnaire (FRQ) [22]. Each item was answered “Yes” or “No” and scored either 0–1 or 0–2, depending on the item. This yielded a maximum total score of 14; higher scores indicated higher risk of falling. A cut-off score of 4 or more indicates high risk of falls in older adults and this threshold was adopted in the present study to classify participants as ‘high fall risk’. The Fall Risk Questionnaire is an extensively validated self-report measure whose items are based on risk factors for falls supported by longitudinal studies. In the original validation study, it demonstrated excellent convergent validity with clinical examination (r = 0.898), and acceptable internal consistency (α = 0.746). The recommended cut-off score had high diagnostic accuracy (AUC = 0.981, sensitivity = 100%, specificity = 83.3%), meeting or exceeding typical benchmarks for screening instruments [22].

#### Functional fitness

To obtain a comprehensive assessment of functional fitness, we combined the protocols of the Senior Fitness Test [23], and the Short Physical Performance Battery [24]. These test batteries are well validated and widely used for the assessment of physical performance in older adults. This approach yielded eight tests designed to evaluate relevant domains of physical performance as follows:

1. 30-Second Arm Curl Test - Evaluated upper limb muscle strength and endurance.
2. 8-Feet Up-and-Go Test - Measured agility and dynamic balance.
3. Back Scratch Test - Evaluated upper-body flexibility.
4. Chair Sit-and-Reach Test - Assessed lower body flexibility.
5. 2-Minute Step Test - Measured cardiorespiratory endurance.
6. Stance Tests (three feet positions: side-by-side, semi-tandem, and tandem stance) - Assessed static balance.
7. 4-Metre Walk Test - Assessed walking speed.
8. Five Times Sit-to-Stand Test - Assessed lower limb muscle strength and power.

All tests were administered according to the description of the developers except for the walk speed, in which we used a 4 m walk test with a dynamic start and stop to account for the effects of acceleration and deceleration [25]. A detailed description of how these tests were administered can be found in the supplementary material (Additional file 1).

#### Exploratory variables

Participant biodata was self-reported. Economic status was evaluated using the question: “How do you manage with the income you have available?”, with response options ranging from “It is easy” to “It is difficult all the time.” Social support was measured using the instrumental support subscale of the Social Support Scale [26], a two-item measure with scores ranging from 2 to 10. Higher scores indicate lower levels of perceived support and a score of > 5 points indicated low support. Pain, anxiety or depression, mobility limitations, and difficulties with activities of daily living were assessed using items from the EuroQol 5 Dimensions 5 Levels (EQ-5D-5 L) instrument [27]. Physical activity and sedentary behaviour were evaluated using two items from the International Physical Activity Questionnaire modified for the elderly (IPAQ-E) [28]: one item assessed walking (volume calculated as frequency × duration), and the other assessed sitting time. Weekly walking durations were categorized as high (≥ 300 min), moderate (150–299 min), or low (< 150 min), in accordance with WHO guidelines for aerobic physical activity [29]. Sedentary behaviour was classified as high (≥ 300 min/day), moderate (181–299 min/day), or low (≤ 180 min/day), based on sample distribution. Cognitive performance was measured using the Six-Item Screener [30], which assesses orientation and memory. Scores range from 0 to 6, with high cognitive performance defined as a score of ≥ 4. Self-reported health conditions were recorded using a checklist adapted from the Australian Longitudinal Study on Women’s Health (ALSWH) [31]. General health and eyesight were self-rated using the questions: “In general, would you say your general health/eyesight is:” with response options ranging from “Excellent” to “Poor.” For analysis, positive responses, i.e. excellent to good were classified as ‘good’ and fair and poor were classified as ‘poor’.

### Statistical analyses

All analyses were conducted in R (version 4.4.2). The overall proportion of missing data was less than 1%, with individual variables containing up to 7.8% missingness. We proceeded under the assumption that data were Missing at Random (MAR) and thus handled missing values using Multiple Imputation by Chained Equations (MICE), generating twenty datasets via Predictive Mean Matching. Analyses were run within each dataset, and results were pooled using Rubin’s rules [32, 33]. For descriptive statistics, we used a completed dataset created by averaging the multiply imputed columns.

Descriptive statistics were used to estimate the prevalence of fall outcomes with 95% binomial confidence intervals. Given that the prevalence of fall outcomes exceeded 10%, and odds ratios from logistic regression could overestimate the association compared to prevalence ratios, we examined associations between fall risk as the independent variable and fall outcomes using Poisson regression with robust variance to obtain prevalence ratios (PRs), adjusting for age, sex, and location.

Similarly, to identify factors associated with fall outcomes, socio-demographic and health-related variables identified in literature [16] were examined using univariable and multivariable Poisson regression with robust variance models. Separate univariable and multivariable models were constructed for each fall outcome (fallers, recurrent fallers, high fall risk) as the dependent variable. Socio-demographic variables included age, sex, residence, and education; health-related variables included musculoskeletal conditions reported, urine incontinence, cognitive performance, pain, anxiety/depression, reported health issues, eyesight, physical activity, sedentary time, and functional limitations. All candidate factors were included in the multivariable models. For recurrent falls, an additional parsimonious multivariable model was constructed due to the limited number of events (low frequency of recurrent fallers) as sensitivity analysis for model stability. This model included only variables significant in the univariable analysis, alongside age and comorbidities as essential covariates. In the results table, we highlight variables whose estimates changed between the full and reduced models. For high fall risk, only socio-demographic factors were assessed.

To explore factors associated with an observed age-patterned distribution of fallers, multivariable logistic regression analyses were conducted separately for participants aged 60–69 years and those aged 75 years and older. Variables included in the models were those that were significant in exploratory univariable analysis.

Population Attributable Fractions (PAFs) were calculated for each Fall Risk Questionnaire item, alongside adjusted odds ratios, to estimate the potential population impact of eliminating established risk factors under the assumption of a causal relationship. This approach does not imply causality within our cross-sectional design, but provides a descriptive measure of the hypothetical reduction in falls if these well-established risk factors, upon which FRQ items are based, were removed.

Associations between fall outcomes (fallers and recurrent fallers) and functional fitness measures were tested using Poisson regression with robust variance, treating each fitness parameter as the independent variable. Three modelling stages were applied: unadjusted; adjusted for age and sex; and fully adjusted for age, sex, location, financial status, medication count, sedentary time, anxiety/depression, and assistive device use. To examine the association between fall risk and functional fitness, fall risk scores were analysed as a count variable using negative binomial regression following a similar three-stage modelling approach. Results of Poisson regressions are presented as Prevalence Ratios (PRs) and that of negative binomial regression as Incidence Rate Ratios (IRRs) with 95% confidence intervals. False discovery rate correction (Benjamini-Hochberg) was applied for multiple comparisons. Multicollinearity was assessed in all multivariable models via Variance Inflation Factor (VIF), with all VIFs < 2.

### Sample size and power

A priori sample size calculation determined that a minimum of 289 participants was required to estimate fall prevalence with ± 5% precision at 95% confidence; the study exceeded this threshold. For regression models, we ensured at least ten events per predictor variable (EPV). There were sufficient observations to support all models except for recurrent falls, where reduced models were used as needed.

## Results

### Participant characteristics

A total of 639 community-dwelling adults aged 60 years and older were included in the study. The mean age was 70.7 years (SD = 7.5). The sample comprised a higher proportion of females (68.9%) and urban residents (60.7%). Detailed sociodemographic characteristics by sex and location are presented in Table 1. Educational attainment varied across the sample. The majority had completed basic education (40.2%); however, females and rural participants were more likely to have no formal or less than basic education (30.5% and 46.0%, respectively) compared to their male and urban counterparts (15.5% and 14.7%, respectively). Most participants were either married (41.0%) or widowed (42.3%). Widowhood was notably more common among Females (53.0%) and rural residents (51.0%). Most participants (79.0%) reported high levels of perceived social support, although low support was more frequently reported by females (23.9%) and rural residents (24.7%).

**Table 1:** Socio demographic profile of participants (*N* = 639)

	Total *n* (%)	Females *n* (%)	Males *n* (%)	Urban *n* (%)	Rural *n* (%)
Age					
60–64	160 (25.0%)	116 (26.4%)	44 (22.1%)	98 (25.3%)	62 (24.7%)
65–69	136 (21.8%)	101 (23.0%)	35 (17.6%)	82 (21.1%)	54 (21.5%)
70–74	163 (25.5%)	102 (23.2%)	61 (30.7%)	99 (25.5%)	64 (25.5%)
75–79	97 (15.2%)	63 (14.3%)	34 (17.1%)	65 (16.8%)	32 (12.7%)
80+	83 (12.9%)	58 (13.2%)	25 (12.6%)	44 (11.3%)	39 (15.5%)
Education level					
No formal education	100 (15.6%)	83 (18.9%)	17 (8.5%)	35 (9.0%)	65 (25.9%)
Less than basic education	65 (10.2%)	51 (11.6%)	14 (7.0%)	22 (5.7%)	43 (17.1%)
Basic Education (10 years)	257 (40.2%)	169 (38.4%)	88 (44.2%)	144 (37.1%)	113 (45.0%)
Secondary School	133 (20.8%)	95 (21.6%)	38 (19.1%)	121 (31.2%)	12 (4.8%)
Tertiary education	84 (13.1%)	42 (9.5%)	42 (21.1%)	66 (17.0%)	18 (7.2%)
Marital Status					
Married	262 (41.0%)	119 (27.0%)	143 (71.9%)	169 (43.6%)	93 (37.1%)
Single/Divorced	107 (16.7%)	88 (20.0%)	19 (9.5%)	77 (19.8%)	30 (12.0%)
Widowed	270 (42.3%)	233 (53.0%)	37 (18.6%)	142 (36.6%)	128 (51.0%)
Household Size					
1–2	167 (26.1%)	124 (28.2%)	43 (21.6%)	96 (24.7%)	71 (28.3%)
3–4	198 (31.0%)	125 (28.4%)	73 (36.7%)	126 (32.5%)	72 (28.7%)
> 4	274 (42.9%)	191 (43.4%)	83 (41.7%)	166 (42.8%)	108 (43.0%)
Economic status					
No difficulties	358 (56.0%)	247 (56.1%)	111 (55.8%)	248 (63.9%)	110 (43.8%)
Some difficulties	195 (30.5%)	125 (28.4%)	70 (35.2%)	106 (27.3%)	89 (35.5%)
Severe difficulties	86 (13.5%)	68 (15.5%)	18 (9.0%)	34 (8.8%)	52 (20.7%)
Engaged in paid work					
Yes	283 (44.3%)	178 (40.5%)	105 (52.8%)	160 (41.2%)	123 (49.0%)
No	356 (55.7%)	262 (59.5%)	94 (47.2%)	228 (58.8%)	128 (51.0%)
Perceived Social Support					
High	505 (79%)	335 (76.1%)	170 (85.4%)	316 (81.4%)	189 (75.3%)
Low	134 (21%)	105 (23.9%)	29 (14.6%)	72 (18.6%)	62 (24.7%)

Basic education is 6 years of primary plus 4 years of middle school. Secondary school is an additional 5/7 years for General Certificate of Education, Ordinary/Advanced level, respectively. Social support was assessed by summing the Likert scores of two questions of Social Support Scale; >5 is low support. Economic status was measured using the question: “How do you manage with the income you have available?”

The health characteristics of participants are summarized in Table 2. Most participants demonstrated high cognitive performance (87.2%) and reported good general health (67.4%). Over one-quarter (28.2%) reported > 5 health issues, and half (50.2%) experienced sleep problems. Mobility difficulties were reported by more than half the sample, difficulties with activities of daily living (ADLs) were less common, with 77.9% reporting none. Pain was widely reported: 56.2% experienced moderate pain, while only 16.1% reported no pain. Symptoms of anxiety or depression were present in nearly one-third of participants. In terms of activity levels, 26.0% exhibited high sedentary behaviour, and over half (54.5%) reported low levels of physical activity.

**Table 2:** Health characteristics of participants

	*n*	%
Cognitive performance		
High	557	87.2%
Low	82	12.8%
Self-rated General Health		
Good	431	67.4%
Poor	208	32.6%
Reported Health Issues		
≥ 5 Health Issues	180	28.2%
< Health Issues	459	71.8%
Sleep Problems		
None	318	49.8%
Some	321	50.2%
Difficulty with Mobility		
None	298	46.6%
Mild	162	25.4%
Moderate/Severe	179	28%
Difficulty with ADLs		
None	498	77.9%
Mild	83	13%
Moderate/Severe	58	9.1%
Pain Experience		
None	103	16.1%
Mild	177	27.7%
Moderate/Severe	359	56.2%
Anxiety/Depression		
None	434	67.9%
Mild	130	20.3%
Moderate/Severe	75	11.7%
Sedentary Behaviour		
High	166	26%
Low	375	58.7%
Moderate	98	15.3%
Physical Activity		
High	168	26.3%
Low	348	54.5%
Moderate	123	19.2%

High cognitive performance = a score ≥ 4 from the six-item screener. Sedentary behaviour: High = ≥ 300, Moderate = 181–299, Low = ≤ 180 min of sitting per day. Physical Activity: High = ≥ 300, Moderate = 150–299, Low = < 150 min of walking per week

### Prevalence of falls outcomes

**Fig. 1:**
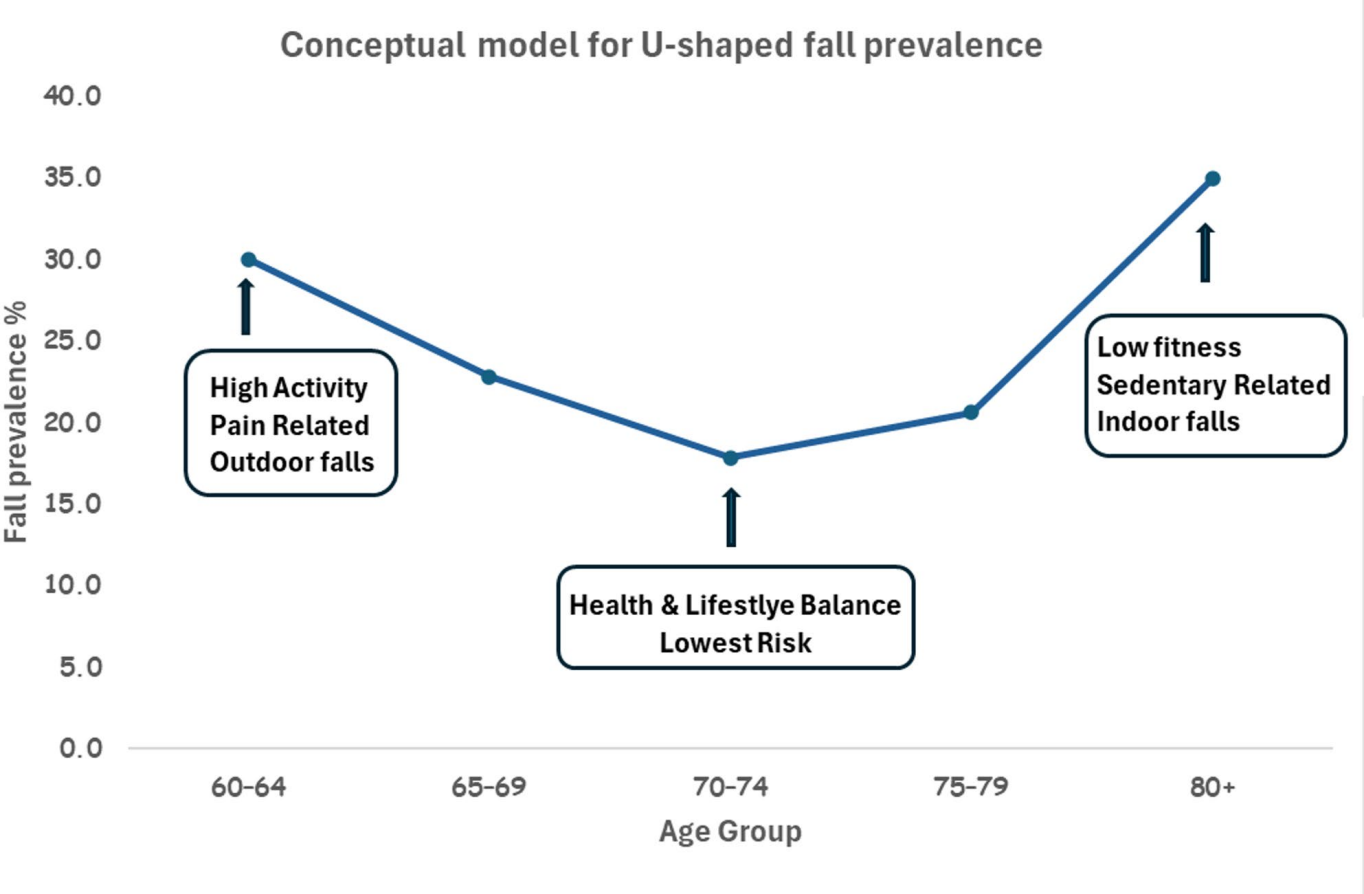
Conceptual model to describe U-shaped fall prevalence among the age groups

Among the 639 participants, 24.6% (95% CI: 21.3–28.1) reported at least one fall in the past year, 10.3% (95% CI: 8.1–13.0) experienced recurrent falls (≥ 2), and 30.2% (95% CI: 26.7–33.9) were classified as having a high fall risk. Complete prevalence estimates stratified by age, sex, and residence are presented in Table 3. A U-shaped prevalence pattern was observed for fallers across age groups (Table 3; Fig. 1). High prevalences were seen in participants aged 60–64 (30.0%) and 80 years and older (34.9%), with the lowest rate recorded among those aged 70–74 years (17.8%). Females and rural residents consistently had higher rates in all fall outcomes than their male and urban counterparts. Among non recurrent fallers, 59.3% reported outdoor falls, and 28.7% of fallers experienced severe falls requiring medical attention.

**Table 3:** Prevalence of fall outcomes by age, sex, and residence

	Total *N* (%)	Fallers % (95% CI), *n*	Recurrent Fallers % (95% CI), *n*	High Fall Risk % (95% CI), *n*	Outdoor Falls; *n* (%)	Severe Falls; *n* (%)
Overall	639 (100)	24.6% (21.3–28.1), 157	10.3% (8.1–13.0), 66	30.2% (26.7–33.9), 193	54 (59.3%)*	45 (28.7%)*
Age Group						
60–64	160 (25.0)	30.0% (23.0-37.7), 48	11.9% (7.3–17.9), 19	24.4% (17.9–31.8), 39	21 (72.4%)	10 (20.8%)
65–69	136 (21.3)	22.8% (16.0–30.8), 31	12.5% (7.5–19.3), 17	25.7% (18.6–33.9), 35	8 (57.1%)	9 (29.0%)
70–74	163 (25.5)	17.8% (12.3–24.5), 29	4.9% (2.1–9.4), 8	26.4% (19.8–33.8), 43	12 (57.1%)	6 (20.7%)
75–79	97 (15.2)	20.6% (13.1–30.0), 20	10.3% (5.1–18.1), 10	35.1% (25.6–45.4), 34	5 (50.0%)	6 (30.0%)
80+	83 (13.0)	34.9% (24.8–46.2), 29	14.5% (7.7–23.9), 12	50.6% (39.4–61.8), 42	8 (47.1%)	14 (48.3%)
Sex						
Females	440 (68.9)	30.5% (26.2–35.0), 134	13.6% (10.6–17.2), 60	36.1% (31.6–40.8), 159	45 (60.8f n%)	38 (28.4%)
Males	199 (31.1)	11.6% (7.5–16.8), 23	3.0% (1.1–6.4), 6	17.1% (12.1–23.0), 34	9 (52.9%)	7 (30.4%)
Residence						
Urban	388 (60.7)	21.4% (17.4–25.8), 83	7.0% (4.6.10.0), 27	22.4% (18.4–26.9), 87	36 (64.3%)	19 (22.9%)
Rural	251(39.3)	29.5% (23.9–35.5), 74	15.5% (11.3–20.6), 39	42.2% (36.0-48.6), 106	18 (51.4%)	26 (35.1%)

* Denominator for outdoor falls in ‘non recurrent fallers’ (*n* = 91), denominator for severe falls is ‘fallers’ (n = 157)

### Association between assessed fall risk and falls

Assessed fall risk was strongly associated with both fallers and recurrent fallers. Participants classified as high fall risk based on the Fall Risk Questionnaire (FRQ) had nearly three-fold higher prevalence of falls (PR = 2.76; 95% CI: 2.08–3.64; *p* < 0.001) compared to those classified as low risk. The association was even stronger for recurrent fallers, with high fall risk individuals having six-fold higher prevalence of recurrent falls (PR = 6.10; 95% CI: 3.47–10.74; *p* < 0.001). When examined as a count variable, higher fall risk scores were consistently associated with greater fall prevalence. Each one-point increase in fall risk scores corresponded to a 20% higher prevalence of previous fallers (PR = 1.20; 95% CI: 1.16–1.25; *p* < 0.001) and a 31% higher prevalence of recurrent fallers (PR = 1.31; 95% CI: 1.24–1.39; *p* < 0.001).

### Socio-demographic and health-related factors associated with fall outcomes

Several socio-demographic and health-related factors were significantly associated with fallers and recurrent fallers in univariable Poisson regression models (Table 4). However, in multivariable models, fewer factors remained significant. Female sex stayed strongly associated with both fallers (PR = 2.67; 95% CI: 1.75–4.09; *p* < 0.001) and recurrent fallers (PR = 3.92; 95% CI: 1.62–9.46; *p* = 0.002). Reporting moderate to severe pain (PR = 1.88; 95% CI: 1.08–3.25; *p* = 0.025), and high sedentary behaviour (PR = 1.41; 95% CI: 1.05–1.88; *p* = 0.021), were also significantly associated with fallers. Interestingly, mild ADL limitations, compared to no limitations, were associated with lower prevalence of fallers (PR = 0.56; 95% CI: 0.34–0.92; *p* = 0.021). Rural residence was the only other factor, besides female sex, which was associated with recurrent fallers. Older age was significantly associated with high fall risk status, but not with fallers or recurrent fallers. In the multivariable model, each additional year of age was associated with a 3% increase in the prevalence of high fall risk status (PR = 1.03; 95% CI: 1.02–1.04; *p* < 0.001). Female participants had nearly two-fold higher prevalence compared with (PR = 1.97; 95% CI: 1.42–2.73; *p* < 0.001). Rural residence was also associated with higher prevalence of high fall risk status (PR = 1.54; 95% CI: 1.19–1.99; *p* < 0.001). The remaining socio-demographic factors were not significantly associated with fall risk.

**Table 4:** Socio-demographic and health-related factors associated with fall outcomes

Variable	Fallers				Recurrent Fallers			
	Univariable PR (95% CI)	*p*-value	Multivariable PR (95% CI)	*p*-value	Univariable PR (95% CI)	*p*-value	Multivariable PR (95% CI)	*p*-value
Age	1.00 (0.98–1.02)	0.717	1.01 (0.99–1.02)	0.589	0.99 (0.96–1.03)	0.658	0.99 (0.96–1.03)	0.753
Sex								
Females	2.63 (1.75–3.97)	**< 0.001**	2.67 (1.75–4.09)	**< 0.001**	4.52(1.99–10.3)	**< 0.001**	3.92 (1.62–9.46)	**0.002**
Location								
Rural	1.38 (1.05–1.81)	**0.020**	1.21 (0.88–1.66)	0.239	2.23 (1.40–3.55)	**0.001**	1.93 (1.09–3.43)	**0.025**
Education status								
None/Less than Basic	1.26 (0.91–1.72)	0.159	1.08 (0.77–1.51)	0.671	2.30 (1.33–3.97)	**0.003**	1.96 (1.07–3.59)	0.028*
Secondary/Tertiary	0.86 (0.61–1.21)	0.381	0.95 (0.68–1.33)	0.758	1.18 (0.64–2.18)	0.587	1.49 (0.80–2.80)	0.212
MSK conditions								
2 + Conditions	2.14 (1.24–3.69)	**0.007**	1.64 (0.94–2.84)	0.079	2.74 (1.02–7.36)	**0.045**	1.99 (0.78–5.09)	0.149
Self-rated Eyesight								
Poor	0.78 (0.50–1.22)	0.281	0.77 (0.50–1.19)	0.236	1.29 (0.70–2.36)	0.416	1.24 (0.68–2.27)	0.489
Cognitive Performance								
Low	1.17 (0.80–1.70)	0.425	0.97 (0.66–1.44)	0.891	0.81 (0.38–1.70)	0.572	0.51 (0.24–1.06)	0.072
Reported Health Issues								
≥ 5 Health Issues	1.26 (0.95–1.68)	0.107	0.91 (0.68–1.21)	0.496	1.55 (0.98–2.48)	0.064	0.99 (0.60–1.64)	0.983
Walking aid								
Yes	0.81 (0.33–1.96)	0.640	0.64 (0.25–1.66)	0.361	1.47 (0.51–4.29)	0.477	1.24 (0.39–3.97)	0.719
Sedentary Behaviour								
Moderate	0.975 (0.65–1.47)	0.905	1.01 (0.67–1.51)	0.976	1.21 (0.64–2.31)	0.546	1.22 (0.64–2.35)	0.545
High	1.31 (0.97–1.77)	0.072	1.41 (1.05–1.88)	**0.021**	1.38 (0.83–2.30)	0.218	1.45 (0.87–2.42)	0.157
Physical Activity								
Moderate	1.41 (0.94–2.13)	0.098	1.42 (0.95–2.12)	0.092	1.49 (0.70–3.15)	0.298	1.37 (0.65–2.87)	0.409
Low	1.26 (0.89–1.79)	0.195	1.00 (0.71–1.42)	1.000	1.66 (0.90–3.08)	0.105	1.14 (0.62–2.11)	0.665
Urine Incontinence								
Yes	1.33 (0.89–1.98)	0.163	1.15 (0.76–1.74)	0.500	1.72 (0.93–3.20)	0.084	1.24 (0.64–2.39)	0.525
ADL Limitations								
Mild ADL	0.73 (0.45–1.18)	0.193	0.56 (0.34–0.92)	**0.021**	0.84 (0.39–1.79)	0.651	0.53 (0.24–1.16)	0.111
Moderate/Severe	1.25 (0.82–1.88)	0.296	0.88 (0.56–1.37)	0.569	1.55 (0.80–2.98)	0.193	0.93 (0.43–2.00)	0.852
Pain								
Mild	1.79 (1.01–3.19)	**0.048**	1.60 (0.90–2.83)	0.107	1.26 (0.49–3.22)	0.628	1.13 (0.47–2.74)	0.786
Moderate/Severe	2.30 (1.35–3.91)	**0.003**	1.88 (1.08–3.25)	**0.025**	2.25 (0.99–5.11)	0.053	1.52 (0.66–3.53)	0.324
Anxiety/ Depression								
Mild	1.16 (0.83–1.62)	0.393	1.03 (0.74–1.43)	0.876	1.30 (0.76–2.24)	0.340	1.02 (0.61–1.72)	0.928
Moderate/Severe	1.48 (1.03–2.13)	**0.036**	1.19 (0.79–1.80)	0.406	1.27 (0.64–2.50)	0.491	0.78 (0.37–1.61)	0.496

Reference categories: Male, Urban, Basic Education, 0–1 MSK (Musculoskeletal) condition, Good Eyesight, High Cognitive Performance, < 5 Health Issues, Low Sedentary Behaviour, High Physical Activity, No ADL (Activities of Daily Living) Limitations, Pain, and Anxiety/ Depression. Categories Definition: High cognitive performance = a score ≥ 4 from the six-item screener. Sedentary behaviour: High = ≥ 300, Moderate = 181–299, Low = ≤ 180 min of sitting per day. Physical Activity: High = ≥ 300, Moderate = 150–299, Low = < 150 min of walking per week

* Not significant in reduced model, *p* values in bold are significant (*p *< 0.05)

Results of multivariable logistic regression analyses that explored the U-shaped distribution of fallers are shown in Table 5. Female sex was strongly associated with fallers in both age groups. Among adults aged 60–69, additional significant factors included having two or more musculoskeletal conditions (OR = 4.42; 95% CI: 1.54–16.23; *p* = 0.012) and any level of pain; moderate/severe pain (OR = 2.97; 95% CI: 1.13–9.37; *p* = 0.040), mild pain (OR = 3.68; 95% CI: 1.29–12.39; *p* = 0.022). Surprisingly, taking 1–2 medications compared with no medication was associated with reduced odds of falls (OR = 0.48; 95% CI: 0.26–0.87; *p* = 0.016). Among participants aged 75 years and older, moderate sedentary behaviour compared to low sedentary behaviour was the additional factor associated with significantly increased odds of falls (OR = 3.59; 95% CI: 1.40–9.42; *p* = 0.008).

**Table 5:** Sociodemographic and health factors associated with fallers: multivariable logistic regression by age group

Variable	OR (95% CI), 60–69 yrs	*p*-value	OR (95% CI), 75 + yrs	*p*-value
Sex				
Females	7.10 (2.98–20.11)	**< 0.001**	4.29 (1.76–11.60)	**0.002**
MSK conditions				
2 + Conditions	4.42 (1.54–16.23)	**0.012**	1.51 (0.50–5.21)	0.485
Sedentary Behaviour				
Moderate	0.66 (0.25–1.60)	0.373	3.59 (1.40–9.42)	**0.008**
High	1.18 (0.59–2.32)	0.631	2.07 (0.88–4.92)	0.094
Physical Activity				
Moderate	1.90 (0.94–3.83)	0.073	1.38 (0.51–3.59)	0.508
High	0.78 (0.37–1.57)	0.491	0.86 (0.32–2.16)	0.754
Medications				
1–2	0.48 (0.26–0.87)	0.**016**	0.89 (0.41–1.96)	0.768
3+	0.45 (0.13–1.38)	0.182	0.89 (0.29–2.63)	0.840
Pain				
Mild	3.68 (1.29–12.39)	**0.022**	0.85 (0.25–2.89)	0.784
Moderate	2.97 (1.13–9.37)	**0.040**	1.14 (0.41–3.46)	0.803

Reference categories: Male, Urban, 0–1 MSK (Musculoskeletal) condition, Low Sedentary Behaviour, High Physical Activity, 0 Medications, No Pain. *p* values in bold are significant (*p *< 0.05)

### Contribution of individual fall risk questionnaire items to fall outcomes

Estimated Population Attributable Fractions (PAFs) are summarized in Table 6. For fallers, the item *“worried about falling”* showed the highest PAF at 24.5% (95% CI: 10.5–38.5), with an adjusted OR of 2.03 (95% CI: 1.38–2.98). Similar contributions were seen for *“pushes with hands to rise from a chair”* (PAF = 24.4%, OR = 1.91) and *“often feels sad or depressed”* (PAF = 15.2%, OR = 1.75). Other items such as *“feels unsteady when walking”* and *“have trouble stepping onto a curb”* showed moderate associations.

**Table 6:** Adjusted odds ratios and Population Attributable Fractions (PAF) for fall risk questionnaire items

FRQ Item Description		Fallers		Recurrent Fallers	
	Prev. (%)	OR (95% CI)	PAF (%) (95% CI)	OR (95% CI)	PAF (%) (95% CI)
Worried about falling	31.6	2.03 (1.38–2.98)	24.5 (10.5–38.5)	3.37 (1.96–5.79)	42.9 (24.0–61.7)
Pushes with hands to rise from chair	35.4	1.91 (1.31–2.80)	24.4 (9.7–39.1)	2.89 (1.68–4.98)	40.1 (20.2–60.0)
Feels unsteady when walking	35.5	1.45 (0.99–2.14)	13.9 (-0.8-28.6)	2.49 (1.46–4.27)	34.7 (14.3–54.9)
Have trouble stepping onto a curb	27.7	1.44 (0.95–2.18)	10.9 (-2.3-24.1)	2.46 (1.40–4.30)	28.8 (9.4–48.1)
Holds furniture when walking	15.2	1.37 (0.84–2.25)	5.4 (-3.8-14.6)	2.56 (1.39–4.71)	19.1 (3.7–34.6)
Often feels sad or depressed	23.8	1.75 (1.15–2.67)	15.2 (2.6–27.8)	1.86 (1.06–3.25)	16.9 (–0.2–34.0)
Uses/advised to use cane or walker	13.6	1.21 (0.72–2.04)	2.8 (-5.4-10.9)	2.40 (1.26–4.55)	16.0 (1.2–30.7)
Loss of feeling in feet	11.6	1.09 (0.62–1.92)	1.0 (-6.0-8.0)	1.63 (0.81–3.26)	6.8 (–4.6–18.1)
Often rushes to toilet	14.4	1.44 (0.87–2.39)	6.0 (-3.3-15.3)	1.48 (0.75–2.91)	6.5 (–6.1–19.1)
Lightheaded/tired from medication	4.4	2.63 (1.19–5.84)	6.7 (-1.3-14.7)	2.10 (0.78–5.61)	4.6 (–3.6–12.8)
Takes sleep or mood medications	10.5	0.96 (0.52–1.77)	-0.4 (-6.6-5.8)	1.14 (0.51–2.57)	1.5 (–8.0–10.9)

All estimates are adjusted for age, sex, and location. CIs computed using the delta method. Prevalence (Prev.) reflects the proportion of participants endorsing each risk item

For recurrent fallers, the most impactful risk factor was again *“worried about falling”* with a PAF of 42.9% (95% CI: 24.0–61.7) and an OR of 3.37. This was followed by *“pushes with hands to rise from a chair”* (PAF = 40.1%), *“feels unsteady when walking”* (PAF = 34.7%), and *“have trouble stepping onto a curb”* (PAF = 28.8%). Other items, such as *“holds onto furniture when walking”*, *“often feels sad or depressed”*, and *“use of a cane or walker”*,* also* contributed meaningfully, with PAFs ranging from 16% to 19.1%.

### Association between falls and functional fitness outcomes

The Mean (± SD) of functional fitness measured by fall status is presented in Table 7. Fallers, recurrent fallers, and participants classified as high fall risk generally demonstrated poorer performance across multiple fitness indicators compared to those without a fall or low fall risk. To assess the association between falls and physical fitness outcomes, we focused on the fully adjusted model presented in Table 8. Comprehensive details of other models are provided in Table 9.

**Table 7:** Mean fitness scores by fall status

Fitness Variable	Overall	Fallers		Recurrent Fallers		High Fall Risk	
	(Mean ± SD)	No (Mean ± SD)	Yes (Mean ± SD)	No (Mean ± SD)	Yes (Mean ± SD)	No (Mean ± SD)	Yes (Mean ± SD)
Stance tests	3.76 ± 0.62	3.76 ± 0.61	3.75 ± 0.64	3.77 ± 0.60	3.72 ± 0.71	3.85 ± 0.44	3.55 ± 0.86
Walk speed (m/s)	1.08 ± 0.30	1.10 ± 0.30	1.04 ± 0.29	1.10 ± 0.31	0.97 ± 0.25	1.16 ± 0.28	0.92 ± 0.29
5x sit-stand (sec)	13.10 ± 4.24	12.93 ± 4.20	13.61 ± 4.36	12.93 ± 4.11	14.56 ± 5.10	12.35 ± 3.43	14.83 ± 5.32
8 Ft up and go (sec)	10.35 ± 3.82	10.21 ± 3.89	10.78 ± 3.56	10.30 ± 3.91	10.79 ± 2.92	9.39 ± 2.36	12.56 ± 5.34
Arm Curls (reps)	15.22 ± 3.71	15.46 ± 3.69	14.48 ± 3.67	15.29 ± 3.72	14.64 ± 3.60	15.63 ± 3.71	14.26 ± 3.54
Chair sit-reach (cm)	5.72 ± 9.03	5.64 ± 9.31	5.97 ± 8.13	5.70 ± 9.02	5.87 ± 9.17	5.80 ± 9.06	5.52 ± 8.97
Back scratch (cm)	-6.87 ± 10.39	-6.83 ± 10.27	-7.02 ± 10.80	-6.76 ± 10.34	-7.89 ± 10.85	-6.30 ± 10.23	-8.20 ± 10.68
2 Min step up (reps)	85.72 ± 23.74	86.65 ± 23.75	82.85 ± 23.56	86.23 ± 23.81	81.24 ± 22.83	89.25 ± 23.60	77.55 ± 22.05

**Table 8:** Association of fitness outcomes with fall outcomes – fully adjusted model

Fitness Variable	Fallers			Recurrent Fallers			Fall Risk		
	PR (95% CI)	*p*-value	*p*-adj	PR (95% CI)	*p*-value	*p*-adj.	IRR (95% CI)	*p*-value	*p*-adj.
Stance tests	0.96 (0.77–1.20)	0.730	0.730	0.94 (0.64–1.36)	0.728	0.728	0.84 (0.75–0.94)	**0.003**	**0.003**
Walk speed (m/s)	0.83 (0.45–1.54)	0.532	0.709	0.32 (0.12–0.90)	**0.033**	0.130	0.36 (0.27–0.47)	**< 0.001**	**< 0.001**
5x sit-stand (sec)	1.03 (1.00–1.07)	**0.027**	0.108	1.08 (1.04–1.13)	**< 0.001**	**0.001**	1.06 (1.04–1.08)	**< 0.001**	**< 0.001**
8 Ft up and go (sec)	1.03 (0.99–1.08)	0.148	0.394	1.01 (0.94–1.09)	0.718	0.728	1.09 (1.07–1.11)	**< 0.001**	**< 0.001**
Arm Curls (reps)	0.95 (0.91–0.99)	**0.021**	0.108	0.96 (0.89–1.03)	0.259	0.346	0.96 (0.94–0.98)	**< 0.001**	**< 0.001**
Chair sit-reach (cm)	0.99 (0.98–1.01)	0.269	0.431	0.98 (0.96–1.01)	0.179	0.323	0.99 (0.98–1.00)	**0.002**	**0.003**
Back scratch (cm)	1.00 (0.98–1.01)	0.638	0.729	0.98 (0.96–1.01)	0.202	0.323	0.99 (0.99–1.00)	**0.015**	**0.015**
2 Min step up (reps)	1.00 (0.99–1.00)	0.210	0.420	0.99 (0.98–1.00)	0.056	0.148	0.99 (0.99–0.99)	**< 0.001**	**< 0.001**

All estimates are adjusted for age, sex, and location, financial status, medication count, sedentary time, anxiety/depression, and assistive device use. p-adj. is p value adjusted for multiple comparison with the False Detection Rate (FDR), *p* values in bold are significant (*p *< 0.05)

**Table 9:** Association of fitness outcomes with fall outcomes – other models

Fallers						
	Model 1			Model 2		
Fitness Variable	PR (95% CI)	*p*-value	*p*-adj.	PR (95% CI)	*p*-value	*p*-adj.
Stance	0.98 (0.79–1.22)	0.865	0.865	1.02 (0.82–1.28)	0.831	0.917
Walk speed	0.62 (0.39–1.01)	0.054	0.145	0.84 (0.47–1.50)	0.534	0.811
5x sit-stand	1.02 (1.00–1.05)	0.074	0.145	1.02 (0.99–1.05)	0.193	0.773
8 Ft up and go	1.02 (1.00–1.05)	0.067	0.145	1.01 (0.98–1.05)	0.454	0.811
Arm Curls	0.94 (0.91–0.98)	0.005	0.042	0.95 (0.91–0.99)	0.009	0.074
Chair sit-reach	1.00 (0.99–1.02)	0.671	0.865	1.00 (0.98–1.01)	0.917	0.917
Back scratch	1.00 (0.99–1.01)	0.860	0.865	1.00 (0.98–1.01)	0.608	0.811
2 Min step up	1.00 (0.99–1.00)	0.090	0.145	1.00 (0.99–1.00)	0.458	0.811

Recurrent Fallers						
	Model 1			Model 2		
Fitness Variable	PR (95% CI)	p-value	p-adj.	PR (95% CI)	p-value	p-adj.
Stance	0.90 (0.64–1.27)	0.553	0.633	0.94 (0.66–1.32)	0.708	0.788
Walk speed	0.29 (0.14–0.60)	0.002	0.008	0.31 (0.13–0.76)	0.013	0.051
5x sit-stand	1.06 (1.02–1.10)	0.002	0.008	1.06 (1.02–1.10)	0.005	0.041
8 Ft up and go	1.03 (0.99–1.06)	0.172	0.293	1.01 (0.96–1.06)	0.701	0.788
Arm Curls	0.96 (0.90–1.02)	0.183	0.293	0.96 (0.90–1.03)	0.241	0.536
Chair sit-reach	1.00 (0.98–1.03)	0.889	0.889	1.00 (0.97–1.02)	0.788	0.788
Back scratch	0.99 (0.97–1.01)	0.436	0.581	0.99 (0.96–1.01)	0.268	0.536
2 Min step up	0.99 (0.98–1.00)	0.102	0.272	1.00 (0.99–1.00)	0.339	0.542

Fall Risk						
	Model 1			Model 2		
Fitness Variable	IRR (95% CI)	p-value	p-adj.	IRR (95% CI)	p-value	p-adj.
Stance	0.74 (0.66–0.83)	< 0.001	< 0.001	0.82 (0.73–0.92)	< 0.001	0.001
Walk speed	0.26 (0.20–0.33)	< 0.001	< 0.001	0.31 (0.23–0.41)	< 0.001	< 0.001
5x sit-stand	1.07 (1.05–1.08)	< 0.001	< 0.001	1.05 (1.03–1.07)	< 0.001	< 0.001
8 Ft up and go	1.11 (1.09–1.12)	< 0.001	< 0.001	1.09 (1.07–1.11)	< 0.001	< 0.001
Arm Curls	0.94 (0.92–0.96)	< 0.001	< 0.001	0.96 (0.94–0.98)	< 0.001	< 0.001
Chair sit-reach	1.00 (0.99–1.01)	0.571	0.571	1.00 (0.99–1.01)	0.413	0.413
Back scratch	0.99 (0.98–1.00)	0.017	0.019	0.99 (0.98–1.00)	0.030	0.034
2 Min step up	0.99 (0.99–0.99)	< 0.001	< 0.001	0.99 (0.99–1.00)	< 0.001	< 0.001

Model 1 is unadjusted. Model two is adjusted for age and sex. p-adj. is *p* value adjusted for multiple comparison with the False Detection Rate (FDR)

For fallers, no fitness measure remained statistically significant following full adjustment and False Discovery Rate (FDR) correction, although all prevalence ratios (PRs) were in the expected direction. Notably, the 5x sit-to-stand time (PR = 1.03; 95% CI: 1.00-1.07; *p* = 0.027) and arm curl repetitions (PR = 0.95; 95% CI: 0.91–0.99; *p* = 0.021) which are measures of muscle strength, showed an association in the fully adjusted model, although neither met the adjusted p-value threshold. Similarly, for recurrent fallers, after full adjustment, the 5x sit-to-stand time (PR = 1.08; 95% CI: 1.04–1.13, p = < 0.001) and walk speed (PR = 0.32; 95% CI: 0.12–0.90; *p* = 0.033) showed associations, however, only the 5x sit-to-stand time remained statistically significant following FDR correction (p-adjusted = 0.001). In contrast, lower performance in all functional fitness measures was significantly associated with increased fall risk (Table 8).

## Discussion

This pioneering study provides one of the first population-based comprehensive assessments of falls, fall risk, and functional fitness among community-dwelling older adults aged over 60 years in Ghana; a country that is representative of the broader sub-Saharan African context. Our findings reveal a pressing public health concern: nearly 25% of participants experienced a fall in the past year, and close to one-third are at high risk of falls. We identified key sociodemographic and health-related factors associated with falls, demonstrated strong associations between fall risk scores and fall history, and established associations between functional fitness and fall outcomes. These results offer critical, population-based evidence in a region where ageing and fall-related research is scarce and lays the groundwork for targeted interventions and policy development to enhance the safety and well-being of the aging population in Ghana and across the region.

### Fall prevalence, demographic patterns, and health factors

The prevalence of fallers (24.6%) and recurrent fallers (10.3%) in our sample align with global estimates [2, 3], and are in line, albeit at the lower end, with regional estimates among community-dwelling older adults, which range from 20% to 55% for fallers and 11% to 20% for recurrent fallers [16].

In our study, female sex and rural residence emerged as consistent sociodemographic risk factors across fall outcomes. The finding that females are at greater risk aligns with systematic reviews [4–6, 16]. Rural residence may reflect environmental hazards which are known risk factors for falls. Furthermore, rural residents in our sample were more likely to have low socioeconomic status, which can be linked to poorer access to healthcare and substandard housing, potentially compounding fall risk. While a similar higher prevalence in rural areas has been reported in South Africa [34], studies from Nigeria suggest a higher prevalence in urban and semi-urban settings [35, 36]. This discrepancy may be an indication of the more predominant location based environmental hazards. Rural areas in low-income countries are often characterized by uneven ground surfaces and inadequate lighting, whereas urban areas may feature crowded streets, heavy traffic, and high pedestrian activity; all of which are hazards that contribute to fall risk [35, 36].

Contrary to established evidence, increasing age was not linearly associated with falls in our sample. Instead, we observed a U-shaped distribution, with higher fall prevalence among the youngest (60–69 years) and oldest (80 + years) cohorts. This pattern suggests distinct mechanisms of falls across the ageing spectrum (Fig. 1). Among younger older adults, falls may be driven by higher physical activity levels and musculoskeletal pain and are therefore more likely to occur outdoors and be less severe. In contrast, falls in the oldest adults appear to be related to reduced functional fitness, age-related decline, and sedentary lifestyles, and are thus more often indoor and more severe. These findings align with other studies reporting U-shaped fall patterns and differing risk profiles for indoor versus outdoor falls in adults aged 65 and older in the United kingdom, United states and China [37–39]. Although age was not directly associated with fall events in our sample, it was significantly linked to increased fall risk classification. This suggests that age contributes more to cumulative vulnerability than to immediate fall occurrence. Overall, these results underscore the need for risk-specific fall prevention strategies and support the use of screening tools that assess broader susceptibility and cumulative risk, rather than relying solely on chronological age or fall history.

Moderate pain and high sedentary behaviour were the health factors associated with increased prevalence of falls, consistent with findings from other sub-Saharan African studies [16]. Interestingly, mild limitations in activities of daily living (ADLs), compared to no limitations, were linked to lower prevalence of falls, potentially reflecting increased caution or compensatory behaviours among those with minor functional impairments.

### Risk of falls

Our findings showed a strong association between self-reported fall risk and falls in past 12 months, indicating the Fall Risk Questionnaire (FRQ) as a useful and practical tool for this population. The FRQ indeed appears to be a simple, scalable screening instrument suitable for use in community settings, including those with limited access to clinical assessments. A similar study in Nigeria using the FRQ supports its applicability in sub-Saharan Africa [35].

Analysis of individual FRQ items highlighted the potentially significant contribution of psychological and functional fitness factors to fall events. Items reflecting concern about falling, anxiety and depression, and impairments in balance and lower limb strength were particularly impactful. These findings suggest that addressing psychological factors and improving strength and balance could significantly reduce fall incidence. Notably, structured exercise programs improve balance, strength, and overall physical fitness and mental health [40, 41], while cognitive behavioural therapy (CBT) has demonstrated efficacy in reducing concern about falling and managing anxiety [42]. Recent umbrella review evidence indicates that combining physical activity interventions with CBT is more effective for community-dwelling older adults than isolated approaches [43]. Importantly, these estimates should be interpreted as hypothetical rather than causal, given the cross-sectional design and the assumptions underlying PAF estimations.

### Functional fitness and fall outcomes

Overall, participants with falls in the past 12 months or high fall risk demonstrated poorer functional fitness. Although many associations did not remain statistically significant after full adjustment and correction for multiple comparisons, the five-times sit-to-stand test emerged as strongly associated with recurrent falls. Other assessments, such as walking speed and arm curls, also showed potential as functional indicators. However, the 8-Ft- Up-and-Go test, a variant of the widely validated Timed Up and Go test [44], did not perform effectively in this context. This suggests that in Ghanaian older adults, measures of muscle strength and walking speed may be more influential in falls management than agility-based measures.

Taken together, the observed patterns, alongside the significant associations between fall risk and all fitness domains; highlight the central role of functional fitness in fall prevention. In particular, the five-times sit-to-stand test stands out as the most robust measure, reinforcing its potential as a simple, practical, and scalable screening tool in low-resource settings.

### Implications for policy and practice

The findings have critical implications for clinical practice and public health in Ghana; an area previously overlooked in public health planning. The high prevalence of falls and fall risk, particularly among women and rural residents, underscores the urgent need for targeted interventions.

Firstly, our findings support the integration of fall risk screening tools, such as the Fall Risk Questionnaire (FRQ), into routine primary care and community health outreach. Secondly, the consistent association between lower limb strength and fall outcomes highlights the value of incorporating simple functional tests, such as the sit-to-stand test, into routine assessments to identify at-risk individuals, guide preventive strategies and monitor rehabilitation progress. Thirdly, the identification of modifiable risk factors such as pain, sedentary behaviour, and concern about falling; offers actionable targets for intervention. Finally, the differing prevalence of falls and risk factors among females, rural residents, and across different age groups underscores the need for targeted interventions that address gender, geographic disparities, and age-specific needs. Policymakers should consider developing national guidelines for fall prevention, incorporating culturally adapted fitness assessments, and investing in training for health workers and caregivers. These efforts would not only reduce fall-related injuries and healthcare costs but also promote healthy ageing and independence among Ghana’s growing older population.

### Strengths and limitations

A key strength of this study is its sampling strategy and population-based design, which included both urban and rural older adults, enhancing the generalisability of findings across diverse settings in Ghana. The use of objective fitness assessments and validated fall risk tools, alongside rigorous training, and quality control procedures, contributed to high data quality.

However, limitations include the cross-sectional design, which precludes causal inference, and reliance on self-reported data may be subject to recall bias, particularly regarding fall history, health conditions, and physical activity and sedentary behaviour. Additionally, the exclusion of individuals with severe impairments may have led to underestimation of fall prevalence in the most vulnerable groups.

## Conclusion

Falls are a significant public health concern among older adults in Ghana, with prevalence and risk patterns shaped by age, sex, residence, and functional status. This study provides foundational evidence to inform fall prevention strategies in sub-Saharan Africa, emphasising the importance of context-specific screening tools and interventions. Future research should explore follow-up and longitudinal outcomes and evaluate the effectiveness of targeted multidimensional programmes to reduce fall risk and promote healthy ageing in the region.

## Supplementary Information

Supplementary Material 1.

## Data Availability

The datasets supporting the conclusions of this article have not yet been deposited in a public repository due to ongoing analysis. They will be made available at a later stage. In the meantime, data are available from the corresponding author on reasonable request.

## Abbreviations

ADL: Activities of Daily Living
ALSWH: Australian Longitudinal Study on Women’s Health
AUC: Area Under Curve
CBT: Cognitive Behavioural Therapy
EQ-5D-5L: EuroQol 5 Dimensions 5 Levels
EPV: Events Per Predictor Variable
FDR: False Discovery Rate
FRQ: Fall Risk Questionnaire
ICC: Intra-Class Correlation Coefficient
IPAQ-E: International Physical Activity Questionnaire modified for the elderly
IRR: Incidence Rate Ratio
LMIC: Lower-Middle-Income Country
MICE: Multiple Imputation by Chained Equations
MSK: Musculoskeletal
OR: Odds Ratio
PR: Prevalence Ratio
PAF: Population Attributable Fraction
SAGE: Study on Global AGEing and Adult Health
SD: Standard Deviation
SSS: Social Support Scale
VIF: Variance Inflation Factor
WHO: World Health Organization
