# Supplementary material for: Falls and functional fitness among older adults in Sub-Saharan Africa: findings from the first population-based cross-sectional study in Ghana
Source: BMC Geriatr. 2026 May 4;26:868. doi: 10.1186/s12877-026-07577-6 (PMC13289247; doi:10.1186/s12877-026-07577-6)
Supplement: Supplementary file 1 — Supplementary Material 1. [file 12877_2026_7577_MOESM1_ESM.docx]

**FRAME-AFRICA DATA COLLECTION SHEET**


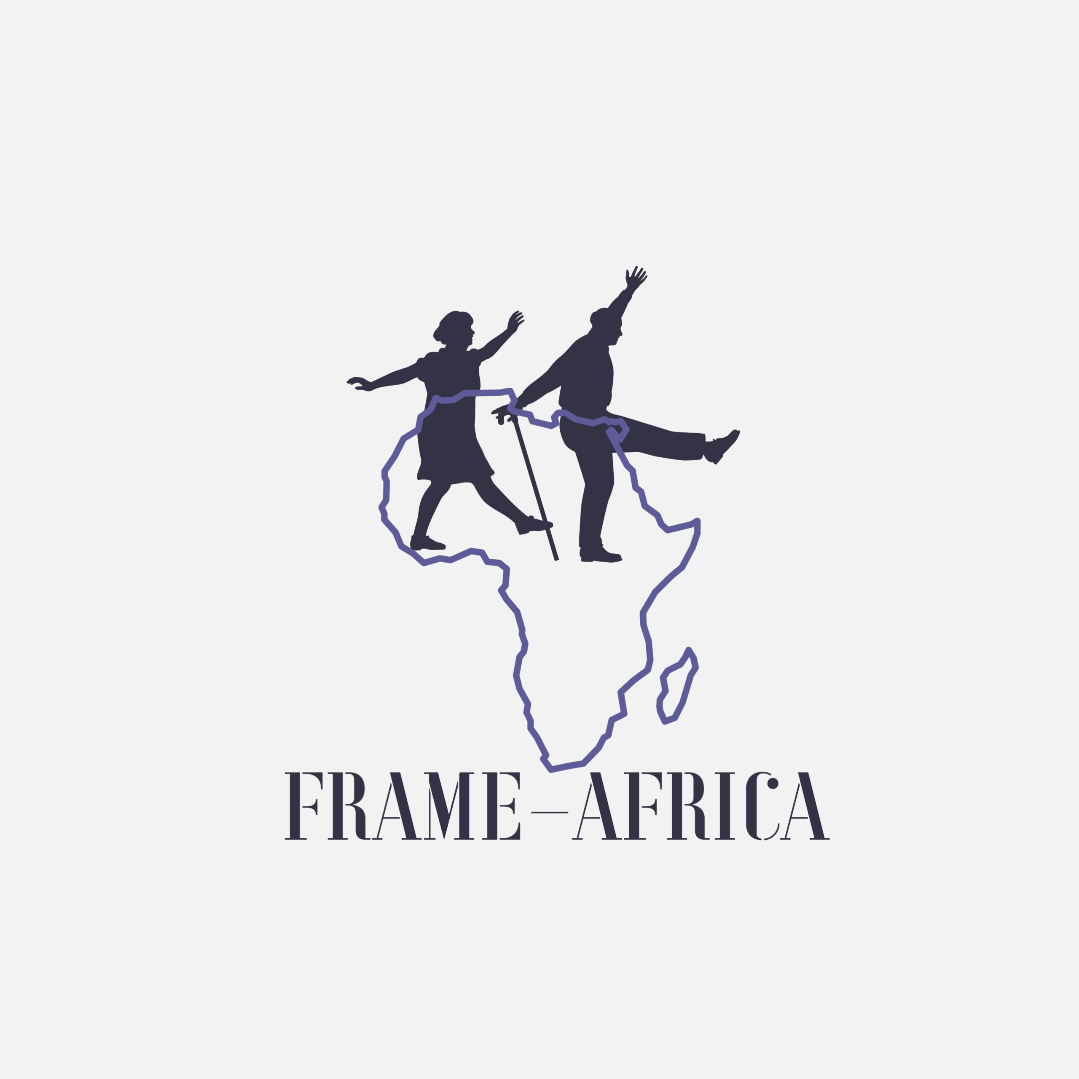


**Participant ID:**

**Location:**

- **Step 1: BIODATA**

| **No.** | **Item** |  | |  | |  | |  | |
| --- | --- | --- | --- | --- | --- | --- | --- | --- | --- |
| **1** | Age: | 60-64 | | 65-74 | | 75-84 | | 85+ | |
| **2** | Sex | M | | F | |  | |  | |
| **3** | Location: | Urban | | Rural | |  | |  | |
| **4** | Level of Education | None | < Basic  ≤ 6yrs | | Basic  10 yrs/ MSLC | | Secondary | | Tertiary |
| **5** | Marital status | Married / Partnership | | Divorced | | Widowed | | Never Married | |
| **6** | Household size: | 1-2 | | 3-4 | | >4 | |  | |

- **Step2: SIX ITEM SCREENER**

**Part A.** Introduce words: **TREE** - **BALL** - **FLAG**.”

- Was task attempted? Yes

No

- If no, why: H = Hearing loss

P = Participant unable to comprehend instructions/cognitive impairment

Other: _________________________

**Part B. Correct Incorrect Unable to answer**

1. What year is this?
2. What month is this?

1. What is the day of the week?

**Part C. Recall**

**Correct Incorrect Unable to answer**

4. TREE

5. BALL

6. FLAG

**SIX result:** ___________

- **Step 3: Social Support and Economic Data**

1. There are people in your life who you can get help with daily activities from if you need it.

Strongly agree Agree Neutral Disagree Strongly Disagree

2. There are people in your life who you can get help with information from if you need it.

Strongly agree Agree Neutral Disagree Strongly Disagree

3. Are you engaged in paid work? Yes No

If yes, what work do you do ________________________________________________________

4. Do you have a steady source of income? Yes No

5. How do you manage on the income you have available?

It is easy It is not too bad It is difficult some of the time

It is difficult all the time It is impossible

6. What is/was your main occupation ___________________________________________________

7. Number of years retired/not working __________________________________________________

- **Step 4: Health Data**

1. Do you have a valid Health insurance? Yes No

2. In general, would you say your health is:

Excellent Very Good Good Fair Poor

3. In general, would you say your eyesight is:

Excellent Very Good Good Fair Poor

4. In general, would you say your hearing is:

Excellent Very Good Good Fair Poor

5. Do you have any of these sleeping problems? (Select all that applies)

Waking up in the early hours of the morning Lying awake for most of the night

Taking a long time to get to sleep Worry keeping you awake at night

Sleeping badly at night None of these problems

6A. Have you lost weight unintentionally in the past 12 months? Yes No

B. How much weight have you lost? ___________________________________________________

C. How did you notice weight loss? ____________________________________________________

D. When did you last check your weight? ___________________________ What was it?_________

7) How often in a week do you feel that everything you do is an effort?

Rarely or none of the time (< 1 day) Some or a few of the time (1-2 days)

A moderate amount of the time (3-4 days) Most of the time (> 4 days)

- **Step 5: EQ-5D-5L**

*For the next set of questions, tell me which statement best describes your health TODAY*

**MOBILITY**

I have no problems in walking about

I have slight problems in walking about

I have moderate problems in walking about

I have severe problems in walking about

I am unable to walk about

**SELF-CARE**

I have no problems washing or dressing myself

I have slight problems washing or dressing myself

I have moderate problems washing or dressing myself

I have severe problems washing or dressing myself

I am unable to wash or dress myself

**USUAL ACTIVITIES** (e.g. work, study, housework, family or leisure activities)

I have no problems doing my usual activities

I have slight problems doing my usual activities

I have moderate problems doing my usual activities

I have severe problems doing my usual activities

I am unable to do my usual activities

**PAIN / DISCOMFORT**

I have no pain or discomfort

I have slight pain or discomfort

I have moderate pain or discomfort

I have severe pain or discomfort

I have extreme pain or discomfort

**ANXIETY / DEPRESSION**

I am not anxious or depressed

I am slightly anxious or depressed

I am moderately anxious or depressed

I am severely anxious or depressed

I am extremely anxious or depressed

*UK (English) © 2009 EuroQol Group EQ-5D™ is a trade mark of the EuroQol Group*

- **Step 6: SPPB**

**1.0 BALANCE TESTS**

**Scoring**:

**1A. Side-by-side-stand**

Held for 10 sec ❒ 1 point

Not held for 10 sec ❒ 0 points

Not attempted ❒ 0 points

Number of seconds held if less than

10 seconds: ____ . ____ sec

**1B. Semi-Tandem stand**

Held for 10 sec ❒ 1 point

Not held for 10 sec ❒ 0 points

Not attempted ❒ 0 points

Number of seconds held if less than

10 seconds: ____ . ____ sec

**1C. Tandem stand**

If participant did not a attempt test or failed, mark why: Which test? ­­­­___________________________

- Tried but unable
- Participant could not hold position unassisted
- Not attempted, you felt unsafe
- Not attempted, participant felt unsafe
- Participant unable to understand instructions
- Participant refused

Other (specify) ________________________________

Held for 10 sec ❒ 2 points

Held for 3 to 9.99 sec ❒ 1 point

Held for < than 3 sec ❒ 0 points

Not attempted ❒ 0 points

Number of seconds held if less than

10 seconds: ____ . ____ sec

**1D.** **Total Balance Tests score _____________**

**2.0 GAIT SPEED TEST (4m)**

**2.A Scoring**

Time for first trial walk ____.____ sec Time for second trial walk ____.____ sec

Walking Aid for walk: ❒ None ❒ Cane ❒Other ______________________

**Average of two trials ____.____ sec**

If participant did not attempt test or failed, mark why:

- Tried but unable
- Participant could not hold position unassisted
- Not attempted, you felt unsafe
- Not attempted, participant felt unsafe
- Participant unable to understand instructions
- Participant refused

Other (specify) ________________________________

If unable to do the walk: ❒ 0 points

If time is more than 8.70 sec: ❒ 1 point

If time is 6.21 to 8.70 sec: ❒ 2 points

If time is 4.82 to 6.20 sec: ❒ 3 points

If time is less than 4.82 sec: ❒ 4 points

**3.0 CHAIR STAND TEST**

**Test chair stand performed:** ❒ Yes ❒ No

**Time to complete five stands ____.___ sec**

**3C. Scoring**

If participant did not attempt test or failed, mark why:

- Tried but unable
- Participant could not hold position unassisted
- Not attempted, you felt unsafe
- Not attempted, participant felt unsafe
- Participant unable to understand instructions
- Participant refused

Other (specify) ________________________________

Failed test stand, or completes stands

in >60 sec: ❒ 0 points

If time is 16.70 sec or more: ❒ 1 points

If time is 13.70 to 16.69 sec: ❒ 2 points

If time is 11.20 to 13.69 sec: ❒ 3 points

If time is 11.19 sec or less : ❒ 4 points

**4.0 Summary Scoring for Complete SPPB**

Total Balance Test score _____ points

Gait Speed Test score _____ points

Chair Stand Test score _____ points **Total Score ______ points**

- **Step 7: Hand Grip Strength assessment**

1.0 Dominant hand Right Left

**Right hand Left hand**

1^st^ Trial . . . . . . . . . . . . . 1^st^ Trial . . . . . . . . . . . . .

2^nd^ Trial . . . . . . . . . . . . . 2^nd^ Trial . . . . . . . . . . . . .

3^rd^ Trial . . . . . . . . . . . . . 3^rd^ Trial . . . . . . . . . . . . .

*Unsuccessful test notes* _______________________________________________________________

- **Step 8: Fall Risk Assessment**

**Part A**

1. Have you fallen in the last 12 months? Yes No

2. How many falls have you had in the last 12 months? _____________ 1 >1

3. Where did you fall? Inside Home Outside Home

4. What activity were you performing when you fell?_____________________________________

5. Were you able to rise on your own when you fell? Yes No

6. Did the fall result in an injury that required attention from a health personnel? Yes No

**Part B: Fall Risk Questionnaire (FRQ)** **YES NO**

1.I have fallen in the last 6 months.*

2.I am worried about falling.

3.Sometimes,I feel unsteady when I am walking.

4.I steady myself by holding onto furniture when walking at home.

5.I use or have been advised to use a cane or walker to get around safely. *

6.I need to push with my hands to stand up from a chair.

7.I have some trouble stepping up onto a curb (a small step).

8.I often have to rush to the toilet.

9.I have lost some feeling in my feet.

10.I take medicine that sometimes makes me feel light-headed or more

tired than usual.

11.I take medicine to help me sleep or improve my mood.

12.I often feel sad or depressed.

**FRQ Score:** ____________

- **Step 9: IPAQ-E**

1. During the last 7 days, how much time did you spend **sitting** during a day?

_______ hours,________ minutes

2a. During the last 7 days, on how many days did you **walk** for at least 10 minutes at a time?

_______ Day (s) or No day

b. How much time did you usually spend walking on one of those days?

_______ hours,________ minutes

3a. During the last 7 days, on how many days did you do **moderate physical activities**?

*These are activities that take moderate physical effort and makes you breathe somewhat harder than normal. Eg: gardening, cleaning, swimming or other fitness activities.* _______ Day (s) or No day

b. How much time did you usually spend doing moderate physical activities on one of those days?

_______ hours,________ minutes

4a. During the last 7 days, on how many days did you do **vigorous physical activities**?

*These are activities that take hard physical effort and makes you breathe much harder than normal like fufu pounding, heavy lifting/gardening, construction work, chopping woods, jogging/running.*

_______ Day (s) or No day

b. How much time did you usually spend doing vigorous physical activities on one of those days?

_______ hours,________ minutes

**Extra question:**

a. Apart from walking what do you do that you consider as exercises? ­­­­­­_________________________

__________________________________________________________________________________

b. During the last 7 days, on how many days did you do **those activities**?

_______ Day (s) or No day

c. How much time did you usually spend doing them on one of those days?

_______ hours,________ minutes

- **Step 10: Comorbidities**

1. **MUSCULOSKELETAL** conditions (check all that applies) None

N

Osteoarthritis Low back pain

Rheumatoid arthritis Other Back pain

Other arthritis Neck pain

Osteoporosis Knee pain

Other joint pain, which joint? ______________________________

N

2. **CARDIOVASCULAR** conditions (check all that applies) None

Heart disease (including heart attack, angina) Thrombosis (a blood clot)

b

Hypertension (high blood pressure) Stroke

N

Hyperlipidaemia Other ________________________

N

3. **NEURODEGENERATIVE** conditions (check all that applies) None

Parkinson’s disease Alzheimer’s disease or dementia

b

Mild Cognitive Impairment (MCI) Other _________________________

N

4. **PULMONARY** conditions (check all that applies) None

Asthma Bronchitis Emphysema

5. **EYE** conditions (check all that applies) None

N

Macular degeneration Cataracts Glaucoma

Other ________________________________

N

6. **PSYCHIATRIC** conditions (check all that applies) None

Anxiety / nervous disorder Depression Other ____________

7. Diagnosed or treated for any **CANCER**? Yes No

B. If yes which type of cancer? _______________________

8. Have you had any **SURGERY**? Yes No

B. If yes which type of surgery? _______________________

**9. Other Conditions** (check all that applies)

Diabetes (high blood sugar) Urinary incontinence

b

Impaired glucose tolerance Severe Headaches

Low iron level (iron deficiency or anaemia) COVID-19

Any other condition ________________________________________________________

**10A. Are you taking any orthodox medications?** Yes No

B. If yes how many different types? _______________________________________________

C. If yes for what reason? _______________________________________________________

**11A. Are you taking any herbal medications?** Yes No

B. If yes how many different types ______________________________________

C. If yes for what reason? _____________________________________________

**12A. How often do you usually drink alcohol?**

I have never drunk alcohol in my life On 1 or 2 days a week

b

I never drink alcohol, but I have in the past On 3 or 4 days a week

I drink rarely On 5 or 6 days a week

b

Less than once a week Every day

B. On a day when you drink alcohol, how many standard drinks do you usually have?

1 or 2 drinks per day 5 to 8 drinks per day

b

3 or 4 drinks per day 9 or more drinks per day

**13. How often do you currently smoke cigarettes or any tobacco products?**

I have never smoked in my life Less often than weekly

b

I have in the past but I never smoke now At least weekly (but not daily)

I smoke rarely Daily

- **Step 11: Seniors Fitness test**

**1.0 8 Feet Up and Go (8FUG)**

Time for first trial _____.____ sec Time for second trial _____.____ sec

Walking Aid for walk: ❒ None ❒ Cane ❒ Other ___________________

*Unsuccessful test notes* _______________________________________________________________

**2.0 30-s arm-curl test of dominant arm**

Number of curls performed ____________________

*Unsuccessful test notes*_______________________________________________________________

**3.0 Chair sit-and-reach test**

First trial

Fingers touched toe = 0 ❒

Fingers did not touch toe (-)_____________

Fingers overlapped (+)_____________

Second trial

Fingers touched toe = 0 ❒

Fingers did not touch toe (-)_____________

Fingers overlapped (+)_____________

*Unsuccessful test notes* _____________________________________________________________

**4.0 Back-scratch test**

First trial

Fingers touched = 0 ❒

Fingers did not touch (-)_____________

Fingers overlapped (+)_____________

Second trial

Fingers touched = 0 ❒

Fingers did not touch (-)_____________

Fingers overlapped (+)_____________

*Unsuccessful test notes* ______________________________________________________________

**5.0 2 min step up test (2MST)**

Record the total number of times the **right knee** reaches the tape in two minutes. ­­­­­­­­­­­______________

Paused to rest? ❒ Yes ❒ No

Held on to a support? ❒ Yes ❒ No

*Unsuccessful test notes* _______________________________________________________________

**6.0** Weight ________________ Height __________________

**Description of Physical Performance tests**

| **TEST** | **PURPOSE** | **DESCRIPTION** | **IMAGE** |
| --- | --- | --- | --- |
| Five times sit to stand (SPPB) | To evaluate the lower limbs’ muscle strength and power. | Time taken to rise and sit back in chair five times. With arms folded around chest. |  |
| 30-s arm-curls (SFT) | To evaluate the upper limbs’ muscle strength | Number of arm curls completed in 30 seconds whiles holding a dumbbell (5lbs for women, 8lbs for men). |  |
| 8-ft up-and-go test (SFT) | To assess agility and dynamic balance | Number of seconds required to get up from a seated position, walk 8 feet (2.44m), turn, and return to seated position. |  |
| Stance tests (SPPB) | To assess static balance | Ability to stand 10sec with feet in tandem, semi-tandem and side-by-side position. |  |
| Back-scratch test (SFT) | To assess upper-body flexibility | Distance between two extended middle fingers when one hand is reaching over the shoulder and the other up the middle of the back. |  |
| 4m (13.1ft) walk test (SPPB) | To assess walking speed | On a 6m meter marked course, participant is asked to walk at their usual pace. Timing begins when a leg crosses the 1m mark and ends at the 5m mark. | 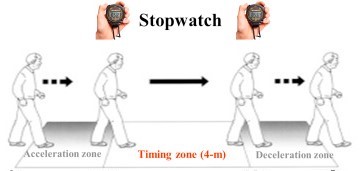 |
| Chair sit-and-reach (SFT) | To assess lower-body flexibility | Distance between two extended middle fingers and tip of big toe when from a sitting position at front of chair, with leg extended the hand is stretched towards toe. |  |
| 2 min step up test  (SFT) | To assess cardio-respiratory endurance | Number of full steps completed in 2 minutes, while raising each knee to a point midway between the patella and iliac crest. | 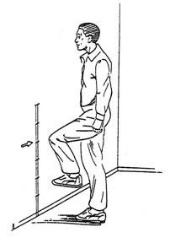 |
| Hand grip strength (FFP) | To assess hand strength using a dynamometer. | With participant seated, the arm under test held at ninety degrees elbow flexion and with the shoulder and wrist in the neutral position. The participants will squeeze the device using maximum force. | 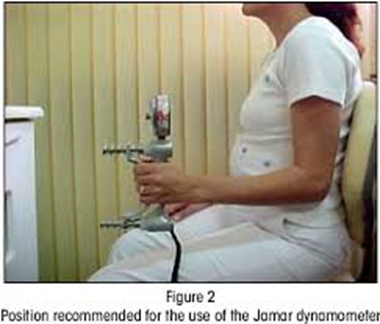 |

SPPB- Short Physical Performance Battery; SFT- Seniors Fitness Test; FP- Fried’s Frailty Phenotype.
